# National Psoriasis Foundation Telemedicine Task Force guidance for management of psoriatic disease via telemedicine

**DOI:** 10.1016/j.jdin.2023.02.018

**Published:** 2023-04-05

**Authors:** Natalia Pelet del Toro, Rayan Yahia, Steven R. Feldman, Abby Van Voorhees, Lawrence Green, Sergio Schwartzman, Evan Siegel, Kelly M. Cordoro, Seemal R. Desai, Leon Kircik, Wilson Liao, Jason E. Hawkes, Jeffrey Weinberg, John Koo, Elizabeth Brezinski Wallace, Leah M. Howard, April Armstrong, George Han

**Affiliations:** a Department of Dermatology, Donald and Barbara Zucker School of Medicine at Hofstra/Northwell, New Hyde Park, New York; b University of California, Riverside School of Medicine, California; c Department of Dermatology, Wake Forest School of Medicine, Winston-Salem, North Carolina; d Department of Dermatology, Eastern Virginia Medical School, Norfolk, Virginia; e Department of Dermatology, George Washington University School of Medicine, Washington, DC; f Department of Rheumatology, Weill Cornell Medical Center, New York, New York; g Arthritis and Rheumatism Associates, Rockville, Maryland; h Department of Dermatology, University of California San Francisco, San Francisco, California; i Department of Dermatology, University of Texas Southwestern Medical Center, Dallas, Texas; j Department of Dermatology, Icahn School of Medicine at Mount Sinai, New York, New York; k Department of Dermatology, University of California San Francisco, San Francisco, California; l Department of Dermatology, University of California Davis Medical Center, University of California Davis Health System, Sacramento, California; m Department of Dermatology, University of Colorado, Aurora, Colorado; n National Psoriasis Foundation, Portland, Oregon; o Department of Dermatology, Keck School of Medicine, University of Southern California, Los Angeles, California

**Keywords:** biologics, psoriasis, psoriatic arthritis, telemedicine, teledermatology, topical therapy

## Abstract

Telemedicine emerged as an alternative care delivery system used to offer effective long-term management to patients with chronic, inflammatory conditions such as psoriatic disease. Teledermatology can provide reliable clinical information through thorough history-taking and virtual evaluations that include patient-provided images and disease activity assessment tools that may help accurately diagnose and manage patients with psoriasis. The integration of validated screening tools for psoriatic arthritis and effective teledermatology practices may improve access to specialists, thus avoiding preventable delays in the diagnosis and treatment of patients with psoriatic arthritis. Although the provision of telehealthcare should not completely replace high quality, in-person dermatologic or rheumatologic visits, the convenience and collaborative nature of teledermatology may lead to expanded access and expedited care in the appropriate setting, whether it be in a virtual or in-person visit.

**Capsule Summary**

• Telemedicine offers more accessible health care to patients with psoriatic disease.
• Virtual evaluations may help accurately diagnose and manage patients with psoriatic disease while expediting care in the appropriate setting, whether it be in a virtual or in-person visit.

## Introduction

The use of technology to provide access to health care is not a new idea, with reports dating back to the 1870s with the use of telephone. Physicians have since continued to leverage emerging technologies to deliver quality care from remote locations.^1^ This was especially apparent when the COVID-19 pandemic caused sudden and drastic changes in health care delivery and access. Telemedicine utilization among dermatologists increased from under 15% to >95% after the pandemic, reflecting its marked popularity among patients and uptake for physicians.^2^^,^^3^ Importantly, this has highlighted that teledermatology can be used beyond the context of a global health crisis to provide continuity of care and improve access to health care more broadly.

## Telemedicine and psoriasis

Teledermatology emerged as an alternative care delivery system used to offer effective long-term management to patients with chronic, inflammatory skin conditions such as psoriasis. Unlike conventional in-person care, accurate diagnosis and management by virtual visits rely partly on the quality of live video images and photographs provided by patients. When properly collected, these images can be of appropriate quality for physicians to make accurate assessments of disease severity using Psoriasis Area and Severity Index (PASI) scores in patients of different skin phototypes.^4^^,^^5^ Alternatively, standardized online training videos may be used to teach patients how to assess PASI, affected body surface area (BSA), and patient global assessment to provide reliable assessments of disease severity when quality images are difficult to produce during virtual evaluations.^6^^,^^7^ Physicians may also guide patients to perform thorough self-evaluations of other areas of their body that might be difficult to appreciate on live video or photographs, such as the scalp and genitals.^8^ Physicians may tailor teledermatology evaluation strategies based on what tools are readily available and accessible to each individual patient.

As compared with in-person care, collaborative and efficient teledermatology models equivalently improve PASI scores, BSA, and dermatology life quality index.^7^^,^^9^^,^^10^ The combination of thorough history-taking and virtual evaluations based on live video or photographs and patient assessments allows dermatologists to manage treatments for psoriasis to achieve significant improvements in disease severity.

Teledermatology expands the ability to deliver patient care. Distance, incapacity, and transportation costs are barriers to in-person care for many patients with psoriasis.^4^ Teledermatology reduces travel costs and time to initiation of care and similarly improves functional status and mental health in patients with psoriasis compared with in-person care.^11^^,^^12^ Teledermatology bypasses physical barriers to care and encourages patients to take active roles in the management of their condition, leading to improved treatment adherence, patient quality of life, and overall health.^9^^,^^13^

Similarly, teledermatology allows physicians to reach patients with psoriasis in institutions with restricted access. Patients with psoriasis who are institutionalized for psychiatric reasons face uncommon difficulties in setting up external appointments and have a high patient turn-around time, in other words, the total time needed for patient transport and completion of an in-patient encounter.^14^ Teledermatology allows comparable management strategies as in-person visits and reduces patient turn-around time by 90% for these patients.^14^ This approach to dermatologic care optimizes patient comfort while providing effective treatment for psoriasis and decreasing the need for chaperoning staff members for in-person evaluations.^14^ Imprisoned patients may also benefit from teledermatology when in-person consults are difficult to coordinate. This may also be used as a tool to address disparities in care provision.^15^ Teledermatology can provide equitable, quality health care to specific subpopulations of patients with psoriasis.

Despite achieving comparable improvements in disease severity, the estimation of areas of involvement and induration of psoriatic lesions are different between teledermatology and in-person evaluations.^16^ Low quality of photos and live video images, lagging internet connection, and lack of an in-person physical examination may contribute to the limited appreciation of certain lesional characteristics in virtual evaluations. Physicians can advise patients to take photos or conduct video visits in well-lit areas with a steady camera set-up to help assure clear, well-focused images for the virtual evaluation. The use of hybrid teledermatology that combines patient-provided photos and a real-time interaction via a telephone call can address issues encountered with a poor internet connection.^17^ BSA determination can be automated, with 96% agreement of the automated photo-based measurement and dermatologists’ BSA measurements.^18^ Teledermatology can allow physicians to efficiently evaluate and manage patients with psoriasis of different skin phototypes and varying disease severity in remote locations.

## Telemedicine and psoriatic arthritis

It is critical to determine if teledermatology is suitable for addressing comorbid conditions that affect patients with psoriasis. Up to 30% of patients with psoriasis develop psoriatic arthritis (PsA); undertreatment and diagnostic delay of PsA lead to irreversible joint damage and significant functional impairment.^19^^,^^20^ Dermatologists may play a key role in mitigating the disease burden by screening patients with psoriasis for joint disease, as 85% of patients develop psoriasis before PsA.^21^ There are 3 validated PsA screening tools available: the Psoriasis and Arthritis Screening Questionnaire, the Psoriasis Epidemiology Screening Tool (PEST), and the Toronto Psoriatic Arthritis Screen. Of these, PEST has strong sensitivity, specificity, and positive and negative predictive values.^22^ PEST is readily accessible for patients on the National Psoriasis Foundation’s website (https://www.psoriasis.org/psoriatic-arthritis-screening-test/), wherein patients can answer 5 simple questions and quickly receive their results for presentation at a virtual evaluation.^23^

Following the identification of suspected PsA, an in-depth discussion and evaluation of joint involvement are warranted. Teledermatology applications using physician-guided palpation, photos of involved joints, and thorough history-taking allow physicians to accurately diagnose active arthritis, dactylitis, or enthesitis; evaluate pain through a visual analog scale; and manage therapeutics for PsA.^24^, ^25^, ^26^ Further physician-guided evaluation techniques involve patients comparing suspected involved digits or joints with the uninvolved contralateral body area, with careful inspection for signs and symptoms of redness, swelling, and pain upon palpation.^8^ In the setting of back pain and stiffness, a thorough evaluation of axial involvement may be conducted through a physician-guided physical examination using specific detailed tests and spinal range of motion tests.^27^

Despite the development of virtual evaluation techniques, the accurate determinations of disease activity may be limited, in some cases of complex joint disease, without an in-person physical examination.^25^^,^^28^ Employing a flexible approach to teledermatology for PsA, in which simpler and established cases are managed through virtual visits and complex cases are followed in an in-person fashion, has been proposed as a solution to provide quality care in the appropriate setting, based on each individual patient’s needs.^29^ Nevertheless, the collaborative approach of virtual evaluations may provide sufficient information to reliably assess for PsA, deliver comparable patient satisfaction to in-person visits based on patient-reported outcomes, and offer prompt management and referrals to a rheumatologist, especially when used to expand access to care when rheumatologists are in short supply.^25^^,^^29^

## Conclusions

Teledermatology is a valuable tool that can be used to supplement other care modalities for patients with psoriatic disease. Although it is not a direct replacement for in-person care, when utilized appropriately, it can be used as a tool to achieve numerous goals of care, such as identifying patients who need a rheumatology referral and increasing access to care. Teledermatology can provide reliable clinical information through thorough history-taking and virtual evaluations that include patient-provided images and disease activity assessment tools that may help accurately diagnose and manage patients with psoriasis. The use of objective measures, including PASI and dermatology life quality index scores, is helpful to quantitively measure patient disease activity but is not necessary for routine clinical care, treatment selection, or assessing treatment response in nonresearch settings. The integration of validated screening tools for PsA and effective teledermatology practices may improve access to specialists, thus avoiding preventable delays in the diagnosis and treatment of patients with PsA. Although the provision of telehealthcare should not completely replace high quality, in-person dermatologic or rheumatologic visits, the convenience and collaborative nature of teledermatology may lead to expanded access and expedited care in the appropriate setting, whether it be in a virtual or in-person visit.

### Position Statements

1. Teledermatology is a reasonable alternative for providing long-term management of patients with psoriasis. Teledermatology may be an option for initial visits, especially when incapacity, distance, and circumstances (eg, access issues among the institutionalized and imprisoned population) prevent the realization of an in-person evaluation.
2. It is important to guarantee the accessibility of in-person care for patients with psoriasis and ensure that telemedicine does not supplant such availability.
3. A discussion about the available virtual evaluation tools (ie, live video visits, patient-provided photos, disease severity assessment tool training videos, etc.) should be undertaken to determine what form of teledermatology is best suited for a patient-physician encounter. This decision should consider patient familiarity with the virtual evaluation platform used, access to stable internet connection, and skillfulness with taking photos and video with electronic devices. Counseling on how to provide high quality images for the encounter should be performed prior to the telehealth visit.
4. Developing better technology—allowing for reliable, high quality image capture and appropriate workflows that emulate a dermatology in-person practice—is important in helping to expand the applicability and utility of teledermatology. Standardized, accessible, and objective modalities to assess BSA/PASI may be helpful in having objective measures to monitor for treatment response. The PEST should be provided to patients every 6 months to screen for PsA to help mitigate the undertreatment and delay in the diagnosis of this common comorbidity in patients with psoriasis.
5. A flexible approach to teledermatology for PsA proposes the efficient utilization of in-person and virtual care based on the severity and complexity of the appreciated joint disease. Thorough history-taking and collaborative evaluation techniques are useful in assessing for joint involvement in patients with suspect PsA. Simpler and established cases may be suitable for virtual evaluations, whereas complex or severe cases may warrant an in-person evaluation and referral to a rheumatologist.
6. Telemedicine has the potential to allow for collaborative connected-health models to expand the reach of experienced dermatologists to help comanage patients with primary care physicians and community dermatologists who are not comfortable with newer systemic treatments for psoriasis.

## Conflicts of interest

Steve R. Feldman received compensation from AbbVie, Accordant, Advance Medical, Almirall, Alovtech, Amgen, Arcutis, Arena, Argenx, Biocon, Boehringer Ingelheim, Bristol Myers Squibb, Caremark, Celgene, Dermavant, Eli Lilly, Galderma, GlaxoSmithKline/Stiefel, Helsinn, Informa, Janssen, Leo Pharma, Menlo, Merck, Mylan, National Biological Corporation, National Psoriasis Foundation Novan, Novartis, Pfizer, Qurient Forte, Regeneron, Samsung, Sanofi, Sun Pharma, Suncare Research, UCB, UpToDate, Valeant, and vTv Therapeutics and is the founder and majority owner of www.DrScore.com and the founder and part owner of Causa Research. Abby Van Voorhees is consultant for Novartis, Celgene, Novartis, Merck, Dermira, UCB, BMS, Amgen, and Boehringer Ingelheim and conducts research for AbbVie, Novartis, and Allergan. Lawrence Green is investigator, speaker, and/or consultant for AbbVie, Amgen Inc, Arcutis, Dermavant, MC2, Novartis, Lilly, OrthoDerm, SunPharma, and UCB. Sergio Schwartzman has received consulting fees, research grants, speakers’ bureau activity, or ownership or partnership from 10.13039/100006483AbbVie, 10.13039/100002429Amgen, Boston Scientifc, Crescendo Bioscience, Dermtech, Eli Lilly, 10.13039/100004328Genentech, 10.13039/100005564Gilead Sciences, Janssen, 10.13039/100004374Medtronic, Myriad Genetics, 10.13039/100003185National Psoriasis Foundation, 10.13039/100004336Novartis, Pfzer, 10.13039/100009857Regeneron, 10.13039/100004358Samsung, Sanof, and UCB and has participated in the advisory boards of Jubilant, Myriad, and Stelexis. Evan Siegel has received research grants, consulting fees, and speaker fees from 10.13039/100006483AbbVie, BMS, Eli Lilly, 10.13039/100004336Novartis, Janssen, and UCB. Kelly M. Cordoro is a founding member of the Psoriasis Investigator Group of the Pediatric Dermatology Research Alliance, a counselor for the International Psoriasis Council, and contributor of psoriasis educational materials to the American Academy of Dermatology. Leon Kircik has received research grants from 10.13039/100006483AbbVie, 10.13039/100007819Allergan, Almirall, 10.13039/100002429Amgen, Arcutis, 10.13039/100001003Boehringer Ingelheim, Breckinridge Pharma, Bristol Myers Squibb, 10.13039/100006436Celgene, Cellceutix, Centocor, Combinatrix, Connetics, Coria, Dermavant, 10.13039/100013988Dermira, Dow Pharma, Dr Reddy’s Laboratories, Eli Lilly, 10.13039/501100009754Galderma, 10.13039/100004328Genentech, 10.13039/100004330GlaxoSmithKline, Idera, 10.13039/100004331Johnson & Johnson, Leo Pharma, Maruho, 10.13039/100004334Merck, Medicis, 10.13039/100004336Novartis AG, 10.13039/100004319Pfizer, PharmaDerm, Promis, Stiefel, 10.13039/501100013671Sun Pharma, UCB, 10.13039/100011284Valeant, and XenoPort and has received honoraria from 10.13039/100006483AbbVie, 10.13039/100007819Allergan, Almirall, 10.13039/100002429Amgen, Arcutis, 10.13039/100006314Biogen Idec, Bristol Myers Squibb, 10.13039/100006436Celgene, Cipher, Connetics, Dermavant, 10.13039/100013988Dermira, Dr Reddy’s Laboratories, Eli Lilly, 10.13039/501100009754Galderma, 10.13039/100004328Genentech, 10.13039/100004330GlaxoSmithKline, 10.13039/100004331Johnson & Johnson, Leo Pharma, 10.13039/100004334Merck, 10.13039/100004336Novartis AG, PharmaDerm, Promis, Serono (10.13039/100004334Merck Serono International SA), Stiefel, 10.13039/501100013671Sun Pharma, Taro, UCB, and 10.13039/100011284Valeant. Wilson Liao has received research grant funding from 10.13039/100006483AbbVie, 10.13039/100002429Amgen, Janssen, Leo, 10.13039/100004336Novartis, 10.13039/100004319Pfizer, 10.13039/100009857Regeneron, and TRex Bio. Jason E. Hawkes has received personal fees/honoraria from 10.13039/100006483AbbVie, Janssen, LearnSkin, LEO Pharma, 10.13039/100004336Novartis, 10.13039/100004319Pfizer, 10.13039/100004339Sanofi, UpToDate, and VisualDx. Jeffrey Weinberg is a speaker for AbbVie, Amgen, Eli Lilly, Novartis, Sun, Leo Pharma, and Pfizer and an investigator for Dermavant, AbbVie, Corrona Psoriasis Registry, Dermira, Lilly, Novartis, and Pfizer. John Koo has served as an advisor for AbbVie, Amgen, Celgene, Janssen, Eli Lilly, Leo Pharma, EPI, Novartis, Pfizer, Sun Pharma, and Ortho Dermatologics, Regeneron/Sanofi, and UCB. Elizabeth Brezinski Wallace serves as an investigator for Pfizer and Target Real World Evidence. April Armstrong is investigator for Sanofi Genzyme, Bristol Myers Squibb, Dermavant, Dermira, Eli Lily, Galderma, Janssen–Ortho, Inc, Kyowa Hakko Kirin, AbbVie, Janssen Pharmaceuticals Inc, Leo Pharma, Modernizing Medicine, Novartis Pharmaceuticals Corp, Ortho Dermatologics, Pfizer Inc, Regeneron Pharmaceuticals, Sun Pharma, and UCB Pharma; a speaker for AbbVie, Regeneron Pharmaceuticals, and Sanofi Genzyme; and on the data safety monitoring board for Boehringer Ingelheim/ Parexel. George Han is investigator, consultant/advisor, or speaker for AbbVie, Amgen, Arcutis, Athenex, Bausch Health, Boehringer Ingelheim, Bond Avillion, Bristol Myers Squibb, Celgene Corporation, Dermavant, DermTech, Eli Lilly and Company, EPI Health, Janssen Pharmaceuticals, LEO Pharma, MC2 Therapeutics, Novartis, Ortho Dermatologics, PellePharm, Pfizer, Regeneron Pharmaceuticals, Sanofi Genzyme, SUN Pharmaceutical Industries Ltd, and UCB and conducts research for Bausch Health, Eli Lilly and Company, Janssen Pharmaceuticals, and Novartis. Natalia Pelet del Toro, Rayan Yahia, Seemal R. Desai, and Leah M. Howard have no conflicts to declare.

## References

[1] Board on Health Care Services, Institute of Medicine (2012).

[2] Kennedy J., Arey S., Hopkins Z. (2021). Dermatologist perceptions of teledermatology implementation and future use after COVID-19: demographics, barriers, and insights. JAMA Dermatol.

[3] Shaikh S., Armbrecht E.S., Kansara V. (2022). Patient and provider experience and outcomes with synchronous teledermatology during the COVID-19 pandemic. JAAD Int.

[4] Singh P., Soyer H.P., Wu J., Salmhofer W., Gilmore S. (2011). Tele-assessment of psoriasis area and severity index: a study of the accuracy of digital image capture. Australas J Dermatol.

[5] Wu D., Lu X., Nakamura M. (2022). A pilot study to assess the reliability of digital image-based PASI scores across patient skin tones and provider training levels. Dermatol Ther (Heidelb).

[6] Armstrong A.W., Parsi K., Schupp C.W., Mease P.J., Duffin K.C. (2013). Standardizing training for psoriasis measures: effectiveness of an online training video on psoriasis area and severity index assessment by physician and patient raters. JAMA Dermatol.

[7] Armstrong A.W., Chambers C.J., Maverakis E. (2018). Effectiveness of online vs in-person care for adults with psoriasis: a randomized clinical trial. JAMA Netw Open.

[8] Gottlieb A.B., Wells A.F., Merola J.F. (2022). Telemedicine and psoriatic arthritis: best practices and considerations for dermatologists and rheumatologists. Clin Rheumatol.

[9] Koller S., Hofmann-Wellenhof R., Hayn D. (2011). Teledermatological monitoring of psoriasis patients on biologic therapy. Acta Derm Venereol.

[10] Chambers C.J., Parsi K.K., Schupp C., Armstrong A.W. (2012). Patient-centered online management of psoriasis: a randomized controlled equivalency trial. J Am Acad Dermatol.

[11] Whited J.D., Datta S., Hall R.P. (2003). An economic analysis of a store and forward teledermatology consult system. Telemed J E Health.

[12] Young P.M., Chen A.Y., Ford A.R. (2023). Effects of online care on functional and psychological outcomes in patients with psoriasis: a randomized controlled trial. J Am Acad Dermatol.

[13] Marasca C., Ruggiero A., Fontanella G. (2020). Telemedicine and support groups could be used to improve adherence to treatment and health-related quality of life in patients affected by inflammatory skin conditions during the COVID-19 pandemic. Clin Exp Dermatol.

[14] Seghers A.C., Seng K.H., Chio M.T. (2015). A prospective study on the use of teledermatology in psychiatric patients with chronic skin diseases. Australas J Dermatol.

[15] Khatibi B., Bambe A., Chantalat C. (2016). [Teledermatology in a prison setting: a retrospective study of 500 expert opinions]. Ann Dermatol Venereol.

[16] Frühauf J., Schwantzer G., Ambros-Rudolph C.M. (2010). Pilot study using teledermatology to manage high-need patients with psoriasis. Arch Dermatol.

[17] Tinio P.A., Melendres J.M., Chavez C.P. (2022). Clinical profile and response to treatment of patients with psoriasis seen via teledermatology during the COVID-19 pandemic in the Philippines. JAAD Int.

[18] Lin Y.L., Huang A., Yang C.Y., Chang W.Y. (2022). Measurement of body surface area for psoriasis using U-net models. Comput Math Methods Med.

[19] Haroon M., Gallagher P., FitzGerald O. (2015). Diagnostic delay of more than 6 months contributes to poor radiographic and functional outcome in psoriatic arthritis. Ann Rheum Dis.

[20] Alinaghi F., Calov M., Kristensen L.E. (2019). Prevalence of psoriatic arthritis in patients with psoriasis: a systematic review and meta-analysis of observational and clinical studies. J Am Acad Dermatol.

[21] Gottlieb A., Merola J.F. (2020). Psoriatic arthritis for dermatologists. J Dermatolog Treat.

[22] Mease P.J., Gladman D.D., Helliwell P. (2014). Comparative performance of psoriatic arthritis screening tools in patients with psoriasis in European/North American dermatology clinics. J Am Acad Dermatol.

[23] Han G. (2021). Psoriatic arthritis diagnosis and management in the era of telehealth. Cutis.

[24] Costa L., Tasso M., Scotti N. (2021). Telerheumatology in COVID-19 era: a study from a psoriatic arthritis cohort. Ann Rheum Dis.

[25] Piga M., Floris A., Congia M. (2022). Telemedicine in rheumatology: high specificity and sensitivity of follow-up virtual video consultations during COVID-19 pandemic. Rheumatol (Oxf Engl).

[26] Leggett P., Graham L., Steele K. (2001). Telerheumatology–diagnostic accuracy and acceptability to patient, specialist, and general practitioner. Br J Gen Pract.

[27] Laskowski E.R., Johnson S.E., Shelerud R.A. (2020). The telemedicine musculoskeletal examination. Mayo Clin Proc.

[28] Kulcsar Z., Albert D., Ercolano E., Mecchella J.N. (2016). Telerheumatology: a technology appropriate for virtually all. Semin Arthritis Rheum.

[29] Chevallard M., Belloli L., Ughi N. (2021). Use of telemedicine during the COVID-19 pandemic in patients with inflammatory arthritis: a retrospective study on feasibility and impact on patient-reported outcomes in a real-life setting. Rheumatol Int.

